# Supplementary material for: Using laboratory data to assess the impact of coronavirus (COVID-19) on reflex cryptococcal antigenaemia (CrAg) testing in South Africa
Source: PLoS One. 2023 Sep 28;18(9):e0292062. doi: 10.1371/journal.pone.0292062 (PMC10538795; doi:10.1371/journal.pone.0292062)
Supplement: S1 File — (PDF) [file pone.0292062.s001.pdf]

UNIVERSITY OF THE WITWATERSRAND, JOHANNESBURG	HUMAN RESEARCH ETHICS COMMITTEE (MEDICAL)

Office of the Deputy Vice-Chancellor (Research and Innovation)

TO: Professor DK Glencross, et al
School of Pathology
Department of Molecular Medicine and Haematology
National Health Laboratory Service

E-mail: Debbie.Glencross@nhls.ac.za

CC: Supervisor: Not applicable
<>
and <HREC-Medical Research Office@wits.ac.za>

FROM: Mr Iain Burns
Human Research Ethics Committee (Medical)
Tel: 011 717 1252

E-mail: Iain.Burns@wits.ac.za

DATE: 2022/02/09

REF: R14/49

PROTOCOL NO: **M220163** (This is your ethics application reference number. Please quote it in all enquiries, oral or written, relating to this study.)

PROJECT TITLE: *Monitoring and analysing laboratory data across the NHLS in support of national programmes (for communicable and non-communicable disease)*

Please find attached the Clearance Certificate for the above project. I hope it goes well and that an article in a recognized publication comes out of it. This will reflect well on your professional standing and contribute to Government funding of the University.

MSWorks2000/Iain0007/Clearscan.wps

UNIVERSITY OF THE WITWATERSRAND, JOHANNESBURG

R49 Professor DK Glencross, et al

HUMAN RESEARCH ETHICS COMMITTEE (MEDICAL)
CLEARANCE CERTIFICATE NO. M220163

NAME: **(Principal Investigator)** Professor DK Glencross, et al

DEPARTMENT: School of Pathology
Department of Molecular Medicine and Haematology
National Health Laboratory Service

PROJECT TITLE: *Monitoring and analysing laboratory data across the NHLS in support of national programmes (for communicable and non-communicable disease)*

DATE CONSIDERED: Ad hoc

DECISION: Approved unconditionally

CONDITIONS: Formerly M17/06/108 (expires on 2022/07/12)

NOTE: If contact information regarding student study participants is required, please contact the Registrar's office - <Nicoleen.Potgieter@wits.ac.za>

SUPERVISOR: Not applicable

APPROVED BY: CB Penny
Dr CB Penny, Chairperson, HREC (Medical)

DATE OF APPROVAL: 2022/02/09

This Clearance Certificate is valid for 5 years from the date of approval. An extension may be applied for.

DECLARATION OF INVESTIGATORS

To be completed in duplicate and **ONE COPY** returned to the Research Office secretariat on the 3rd floor, Phillip Tobias Building, Parktown, University of the Witwatersrand, Johannesburg.

I/we fully understand the conditions under which I am/we are authorized to carry out the above-mentioned research and I/we undertake to ensure compliance with these conditions. Should any departure be contemplated from the research protocol as approved, I/we undertake to submit details to the Committee. **I agree to submit a yearly progress report**. When a funder requires annual re-certification, the application date will be one year after the date when the study was initially reviewed. In this case, the study was initially reviewed in **January** and therefore reports and re-certification will be due in the month of **January** each year. Unreported changes to the study may invalidate the clearance given by the HREC (Medical).

_______________________ _______________________
Signature of Principal Investigator Date
