# Supplementary material for: Using laboratory data to assess the impact of coronavirus (COVID-19) on reflex cryptococcal antigenaemia (CrAg) testing in South Africa
Source: PLoS One. 2023 Sep 28;18(9):e0292062. doi: 10.1371/journal.pone.0292062 (PMC10538795; doi:10.1371/journal.pone.0292062)
Supplement: Attachment — Submitted filename: PONE-D-22-31762 Author changes made 19 05 2023.docx [file pone.0292062.s002.docx]

**Reviewer Comments**

**Reviewer One**

Reviewer #1: On the whole, a well-written article in terms of structure and content.

The research question seems to be along the lines of "What was the impact of COVID-19 on CrAg testing in South Africa?".

| **Reviewer Comments** | **Author Response** |
| --- | --- |
| Only three issues were detected on language review: | Agreed, changes made to the manuscript. |
| 1. "one" instead of "on" p. 4 | Agreed, changed made to text. Revised to ‘Provider-initiated CrAg testing is done on request by a clinician/ health care worker and done through microbiology laboratories of the NHLS’ |
| 2. "curve" instead of "curb" p. 13 | Agreed, change made. Revised to ‘It has been argued that strict lockdown measures implemented to curb the spread of SARS_COV-2 infections could have long-lasting public health ramifications that transcend the immediate health risks posed by COVID-19’ |
| 3. It should be made clear that "CrAg positivity" and "CrAg detection" are synonymous (if I understand correctly). | Page 5 – inserted text (Lines 152-153):  ‘For this manuscript, the terms CrAg positivity and detection are synonymous.’ |
| 47 vs. 49 labs? Is it the case that there are 49 CD4 labs, only 47 of which do reflex CrAg testing? | Apologies, this was a typo – we only have 47 CD4 laboratories that also offer reflexed CrAg testing.  Text amended on page 5:  ‘Context: Data is reported for all CrAg testing performed across 47 CD4 laboratories within the South African National Health Laboratory Service (NHLS), the largest diagnostic pathology service in the country supporting the national and provincial health departments’ |
| Under "Methods: Statistical Analysis" monthly test volumes are stated to be compared to 2019. In "Results: Percentage change..." it is stated to be compared to the respective month in 2018 - this is confirmed by the legend in Figure 1. Please clarify/correct. | Agreed, this is an error. The Methods have been updated to indicate that the percentage change is compared to the respective month in 2018.  Amended text on Page 6:  ‘The monthly test volumes for 2020 and 2021, when lockdown levels were in place, were compared to the corresponding month in 2018 (pre-COVID-19); the percentage change or difference is shown as a line chart, e.g., percentage change between January 2020 and 2018.’ |
| "A p-value of ≤0.001 was reported for the chi-squared test for the association between the calendar year and CrAg results" - does this imply that statistical significance was found in the trends for both a) CrAg volumes and b) CrAg positivity? | Agreed, there was a statistical significance found. Both the methods, results and table have been amended.  Amended Methods:  ‘The chi-squared test was used to assess the association between calendar year and CrAg result (positive or negative), assuming an alpha of 0.05’  Amended Results:  A p-value of ≤0.001 was reported for the chi-squared test for the association between the calendar year and the CrAg result indicating a statistically significant difference.  Amended Table 1:  Table 1: Reflexed cryptococcal antigenemia (CrAg) test volumes for samples with a CD4 count <100 cells/µl received within the National Health Laboratory Service for calendar years 2018 to 2021. The number of CrAg positive samples is also reported to determine the annual CrAg positivity across South Africa. The chi-squared test was used to assess the association between calendar year and CrAg result (positive or negative), assuming an alpha of 0.05   |
| There doesn't seem to be statistical significance determination for the CrAg volumes during the discrete waves, nor for the CD4 median/categories, even though the conclusion in the abstract notes "A smaller impact on the median CD4 was noted" - what was the magnitude of the impact, and was it significant? The conclusion in the body of the manuscript seems to allude to significance by saying "...lower median CD4 counts during the respective COVID-19 waves". From a birds-eye (non-statistical perspective), the lack of change observed in distribution of CD4 categories would argue against a significant change. | Agreed, we have now done a median analysis of the CD4.  Added to Methods:  ‘We assessed whether the monthly median CD4 (for a count <100 cells/µl) and CrAg positivity differed for two periods defined as pre-COVID-19 (January 2018 to February 2020) and COVID-19 (March 2020 to December 2021).’  Added to Results:  ‘The monthly median CD4 reported a skewness of -1.330031, hence the non-parametric Mann Whitney test was used (data reported for 48 observations with the exact option used), The results indicate that there was no significant difference between the monthly median CD4 for the two groups (pre-COVID-19 and COVID-19) with a p-value of 0.7431. However, a significant difference was noted for CrAg positivity with a p-value of 0.021.’ |
| The is a lot of data presented about the provincial percentage changes, however, the narrative is quite sparse. Did you have a hypothesis prior to analysing this data, and how did the findings relate to the hypothesis? | Agreed, we have added some narrative to the Methods section to describe why the provincial analysis was done.  Text added:  ‘Local studies have shown that the percentage of samples with a CD4<100cell/µl and viral suppression (<400 copies/ml) varied per province[24, 25] NHLS annual reports from 2018-2022 confirmed the range of provincial CD4%<100 cells/µl between 3.1-27% with little variability between years.[20, 26-28] The annual percentage CD4 samples with a count <100cells/µl was stable at 10% with a positivity of 6.2% between 2018-2022.[20, 26-28]’  ‘ |
| It may be useful in figures 2 and 3 to indicate the COVID-19 waves or lockdown periods (e.g. shading of relevant months). | Agreed, the shading of the relevant months for the COVID-19 waves have now been added to Figures 2 and 3.  Amended figure legend and figures:  Amended Figure 2 Legend:  Figure 2: Analysis of the monthly median CD4 for a count <100 cells/µl for calendar years 2018 to 2021 The reflexed cryptococcal antigenemia (CrAg) positivity is indicated on the secondary y-axis. Data is reported for testing received within the National Health Laboratory Service. The SARS CoV-2 waves, from one to four, as defined by the National Institute for Communicable Diseases (NICD) are depicted as blue bars.  Amended Figure 2:    Amended Figure 3 Legend:  Figure 3: Analysis of the monthly percentage of samples with a CD4 count <25, 25-50, 51-75 and >75-<100 cells/µl for reflexed cryptococcal antigenemia (CrAg) testing performed for calendar years 2018 to 2021. The CrAg positivity is indicated on the secondary y-axis. Data is reported for testing received within the National Health Laboratory Service. The SARS CoV-2 waves, from one to four, as defined by the National Institute for Communicable Diseases (NICD) are depicted as blue bars.  Amended Figure 2:   |
| The conclusion mentions "This confirms that patient CrAg testing overall was more affected by COVID-19 than CD4 testing." It is acknowledged in the earlier paragraph of the manuscript that the CD4 test volume data is not shown, but I think either the authors should show/reference the data, or remove mention of this. | Agreed, this data is now shown clearly in Table 4. |
| "Another limitation of this study was that no linking of the CrAg and COVID-19 results" - would this really have been a valuable analysis to undertake given the differing incubation periods of COVID-19 and cryptococcosis? | Agreed, we have added this as a limitation.  ‘A further limitation of this study is that there was no linking of the CrAg and COVID-19 results to develop a cohort due to the absence of a unique patient identifier. This cohort analysis would have been generated very valuable insights given the differing incubation periods for COVID-19 and cryptococcosis.’ |
| I feel like there are more limitations than acknowledged: | Agreed, we have responded to each point raised below. |
| - Was the reflex testing cohort chosen for convenience purposes? Although the non-reflex CrAgs probably do not contribute to a large number of tests, the selection of reflex only could introduce bias. | Agreed, text added to limitations:  ‘Data is only reported for reflex CrAg testing, which is line with local guidelines.[19] The omission of provider-initiated CrAg testing may introduce some bias. However, a local study reported very high coverage of 95% for reflex screening which indicates that any potential bias by omitting provider-initiated data may be very limited.[38]’ |
| It is not clear why a lower limit of 15 years was chosen for the sample. Guidelines for CD4 +/- CrAg include all adolescents from age 10 years and above, as far as I am aware of. Is this also related to convenience? | We agree, unfortunately for this study data was only requested for those 15 years and older. The following text have been added to limitations:  ‘Another limitation was that data was only extracted for testing for those aged 15 years and above. The decision to exclude the younger ages was based on the weekly surveillance data generated by NICD that reported that the cumulative number of Covid-19 cases from 3 March 2020 to 1 January 2022 was 93% for 15 years and older.[39] An additional analysis may be considered to include those aged 10 to 14 years.’ |
| - The discussion does not take into consideration that there was a change in testing strategy implemented in 2019/2020. Most notably with newer guidelines, 6-monthly CD4 testing is indicated if VL > 1000. So although surveillance CD4 from the "wellness pre-ART" cohort is no longer a consideration, we could expect an increase in CD4 testing in 2019/2020 (compared to 2018) due to more frequent testing of patients who fail to achieve virological suppression. | Agreed, we have added text to the discussion:  ‘However, guideline changes implemented in 2019/20 would have resulted in CD4 testing for those with an HIV viral load >1000 copies/mL as part of ART monitoring most likely would likely resulting in an increase in CD4 test volumes [29] However, our findings report dramatic reduction in CD4 testing of between for 2020 and 2021.’ |
| Regarding non-availability of data due to lack of permission, why is this the case? What steps were taken to gain permission to share the data as part of the proposed publication? | Agreed, we have now made this data fully available as a new Table. The  Added text to Methods (Page 6):  ‘Annual CD4 test volumes, irrespective of the count, were assessed to determine the percentage change between 2018 and 2021. This was then contrasted to the percentage change in reflexed CrAg testing for a count <100 cells/µl.’  Added text to Discussion:  ‘Overall, a -3.3% percentage change in test volumes was reported between 2018 and 2019 (Table 4). However, for 2020 and 2021 this increased to -14.3% and -5.8% demonstrating that COVID-19 had a significant impact on test volumes (despite the downward trend of ~3% due to guideline changes removing the need for CD4 testing for ART eligibility).[26] The findings also show that a much higher percentage change in reflexed CrAg testing was noted for 2020 and 2021 when compared to CD4 testing.’  Added a new Table 4:  Table 4: Analysis of CD4 test volumes for samples received within the National Health Laboratory Service for calendar years 2018 to 2021, South Africa. The annual percentage change in test volumes is also indicated. The percentage change for reflexed CrAg testing was also reported.   |
|  |  |

**Author changes:**

**Author Change**

Table 3 – amended age category captured as 50-49 to the correct value: 50-59 (typo)

Minor grammatical changes.

Amended figures 2 and 3 (updated TIFF files are attached).

Added text to the data availability statement:

‘Data is available from the National Health Laboratory Service Corporate Data Warehouse, subject to an application to the Academic Affairs and Research Office. This application can be made using the online system (<https://aarms.nhls.ac.za/>).’
