# Supplementary material for: Using laboratory data to assess the impact of coronavirus (COVID-19) on reflex cryptococcal antigenaemia (CrAg) testing in South Africa
Source: PLoS One. 2023 Sep 28;18(9):e0292062. doi: 10.1371/journal.pone.0292062 (PMC10538795; doi:10.1371/journal.pone.0292062)
Supplement: Attachment — Submitted filename: PONE-D-22-31762 Author changes made R2 29 08 2023.docx [file pone.0292062.s003.docx]

**Editorial Changes**

| **Comment** | **Author response** |
| --- | --- |
| 1. Please correct the language issues i.e. line 140 do you mean date or data | We have reviewed the sentence and have kept as date. Text has amended to indicate that we are referring to the sample reviewed date.  ‘The sample reviewed date is electronically generated by the laboratory information system (LIS) when CD4 and CrAg results are verified and released.’  Amended text in line 142:  ‘The sample reviewed date was used to extract the year and month for each episode number.’ |
| 2. Please explain how samples from the same patient were handled? | Agreed, added text:  ‘This a specimen-level data extract and the data was not deduplicated due to the absence of a unique patient identifier. Hence, our data may include multiple results per patient. An earlier study that used de-duplicated patient data reported no significant differences in CrAg positivity, indicating that specimen-level data was sufficient.[21]’ |
| 3. Your table and figure headings are too long. Please ensure that they are short and succinct. | Agreed, table and figure heading have been amended as recommended.  Revised table and figure headings:  ‘Table 1: Annual CD4 and CrAg test volumes are reported, with the percentage contribution indicated. The CrAg contribution to CD4 test volumes and positivity are also reported. The percentage change is relative to 2018 data.’  ‘Table 2: Analysis of gender and age category distribution for positive reflexed CrAg test volumes per calendar year, with reference to positivity rates.’  ‘Figure 1: The monthly percentage change in reflexed CrAg test volumes for 2020 and 2021 were compared to 2018 data. The number of SARS CoV-2 cases are reported as bar charts, with waves indicated.’  ‘Figure 2: Monthly median CD4 values for a count <100 cells/µl for 2018 to 2021 are plotted against the reflexed CrAg positivity.’  ‘Figure 3: Monthly percentage with a CD4 count <25, 25-50, 51-75 and >75-<100 cells/µl are reported for 2018 to 2021. The CrAg positivity is also indicated.’  ‘Table 3: Provincial annual percentage change in reflexed cryptococcal antigenemia (CrAg) test volumes for calendar years 2020 and 2021, compared to the correlating data reported for 2018. The provincial reflexed cryptococcal antigenemia (CrAg) positivity is also reported.’ |
| 4. When describing data using the monthly median values it is standard practice to include the IQR. | Yes, it is the preferred method to report an interquartile range for median values (Habibzadeh F. Statistical Data Editing in Scientific Articles. J Korean Med Sci. 2017 Jul;32(7):1072-1076. doi: 10.3346/jkms.2017.32.7.1072. PMID: 28581261; PMCID: PMC5461308.) |
| 5. You conclusion should be renamed discussion and the conclusion should be short and succinct also covering how this study impacts practice. | Agreed, we have revised the manuscript and added a discussion and conclusion.  Revised Discussion:  ‘Discussion  This study aimed to assess the impact of COVID-19 on cryptococcal disease in South Africa. Aggregate CD4 test volumes showed a significant reduction in 2020 and 2021 compared to 2018. Overall, a declining trend in testing of around 5% per annum was noted pre-COVID-19 due to revised HIV guidelines of “test and treat” introduced in 2016.[19, 27, 28] However, the impact of COVID-19 exacerbated the test volume reductions for 2020 and 2021. Of greater concern was that the impact on reflexed CrAg testing was even more substantial. The proportion of samples with a count <100 cells/µl did not change substantially over the study period, indicating that the reduction in CrAg test volumes followed trends in CD4 tests.  National CrAg positivity increased by one percent in 2020. Historical data reported a detection range of 4.2% to 5.6% between 2015 and 2017. This highlights that the CrAg positivity in 2020 is incongruent with the pre-COVID-19 annual trends. By 2021, the national CrAg positivity reduced to levels reported before 2020, despite lower CrAg volumes and less restrictive levels imposed, confirming that higher lockdown levels imposed in 2020 resulted in higher detection rates.  While reflex CrAg test volumes reduced substantially, there were no significant demographic changes noted (age and gender). Across the study period, more males received a CrAg result, most likely related to well-described late presentation of males to clinics locally.[29-31] Similarly, the majority of testing was for ages 20 to 49 which is similar to routine CD4 testing. Therefore, it does not appear that the lockdown restrictions impacted the age and gender composition of those seeking healthcare where CD4 testing was requested.  The monthly analysis for reflexed CrAg testing (<100 cells/µl) showed that during COVID-19 wave one to four, a reduction of up to -36.6% was reported compared to 2018 data, confirming that CrAg volumes were substantially affected by COVID-19 outbreaks.  As CrAg rates of detection peaked in 2020 during the first wave of infections, the median CD4 for a count <100 cells/µl reduced from 43 cells/µl to 39 cells/µl (count <100 cells/µl) highlighting that patients were more immune-suppressed. i.e. presented later. Subsequently, the median CD4 recovered to levels reported before 2020. In contrast, the distribution of CD4 categories was relatively unaffected by the pandemic. Later presentation indicates that severely ill patients were the ones presenting for care. Data reported thus confirms that the increase in CrAg positivity during 2020 was associated with a median CD4 decline.  Local studies have shown that the percentage of samples with a CD4<100cell/µl and viral suppression (<400 copies/ml) varied per province[29, 32] NHLS annual reports from 2018-2022 confirmed the range of provincial CD4%<100 cells/µl between 3.1-27% with little variability between years.[33-36] The annual percentage CD4 samples reported with a count <100cells/µl was stable at ~10% with a positivity of 6-7% before 2018.[33-36] Given the disparity in both very advanced HIV disease and viral suppression previously reported, we assessed provincial CrAg test volumes and positivity for each calendar year of the test period.  The provincial analysis revealed that CrAg test volumes decreased in 2020 and 2021 except for the Northern and Western Cape provinces. In particular, provinces such as the Free State, Gauteng, KwaZulu-Natal and Mpumalanga reported substantial reductions for 2020 exceeding twenty percent. However, for 2021, further reductions of over thirty percent were noted for four provinces, while this trend was only noted in one province in 2020. These findings indicate that lockdown restrictions may have resulted in reduced access as many health facilities closed, especially during levels four and five.[11, 12] The Western Cape was the only province where an increase was noted in CrAg test volumes for 2020 and 2021 relative to previous years. It is not possible to determine the reason for this from the data presented here as it may be multi-factorial. One possibility could be the lower HIV prevalence reported for the Western Cape Province.[37]  Overall, the highest CrAg detection rate was reported for the KwaZulu-Natal province, confirmed in earlier studies, even though they reported the lowest percentage CrAg tests of total CD4 tests conducted.[23, 38] However, between 2018 and 2020 the detection rate increased in all nine provinces, which is out of the norm of reported historical CrAg positivity data showing stability over time at provincial level. Additional analysis of CrAg positivity recovery rates at district, sub-district and facility levels is needed to understand the changes reported during COVID-19, particularly in 2020.  It has been argued that strict lockdown measures implemented to curb the spread of SARS_COV-2 infections could have long-lasting public health ramifications that transcend the immediate health risks posed by COVID-19.[11, 12, 39] The study findings suggest that patients may have delayed seeking HIV care due to several factors including the COVID-19 crisis, overloaded hospitals, limited access, and fear of exposure.[39] The lockdown also resulted in job losses for formal and informal workers.[39] A local study reported that the South African growth domestic product (GDP) was expected to reduce by between 5 and 10 percent during 2020, due to significant job losses reported.[39-41] In times of crisis and loss of income, communities are more likely to prioritise food, water, safety and shelter rather than traveling to a health facility, and this may be indicative of why these patients with very advanced HIV disease did not present for care sooner.[13] This supports the notion that a sustained lockdown has had a prolonged, longer-term negative impact on patients accessing care and receiving testing. Similar findings in respect of restricted access to care and reduced testing for tuberculosis (TB) have been reported.[13] These findings are in line with WHO reporting, that for the first time, reported a TB-related mortality increase in 2020.[42]’  Revised conclusion:  ‘Conclusion  In conclusion, our findings revealed that there was a negative impact of COVID-19 on the absolute numbers of CrAg tests performed, as well as an increase in detection rate in certain provinces, probably reflecting only severely ill patients with very low CD4 counts, presenting themselves for care. The work highlights the missed opportunity for screening patients with advanced HIV for Cryptococcal disease during the COVID-19 pandemic in South Africa. This work provides evidence of the usefulness of secondary laboratory data to investigate the impact of pandemics on national laboratory testing services.’ |
| 6. Please do not repeat results in discussion. Rather highlight main finding and discuss them. | Agreed, results have been removed from Discussion. |
| 7. Table 4 belongs to results | Agreed, this has incorporated into Table 1 |
| 8. Please also include the health department ethical approval/ permission in your submission. | Agreed, HREC certificate uploaded on the submission sites |

**Author changes:**

**Author Change**

The manuscript has been reviewed and revised by the authors. These changes are described below. Minor editing are not described.

Amended study objectives:

‘The primary objective of the study was to assess the impact of COVID-19 on cryptococcal disease test volumes and burden (positivity). Secondary objectives set out to establish the impact on demographics (gender and age) of CrAg tests performed.’

Amended Table 1 to indicate CD4 volumes:

Changes made to Methods:

Data preparation:

‘Aggregate CD4 volumes irrespective of count were extracted by calendar year and month.’

Statistical analysis:

‘Annual CD4 test volumes, irrespective of the count, were assessed to determine the percentage change between 2018 and 2021. This was then compared to the percentage change in reflexed CrAg testing for a CD4 count <100 cells/µl. The contribution of reflexed CrAg to CD4 test was assessed.’

Changes made to Results:

‘Over the four year test period, a total of 11.9 million CD4 tests were performed across 47 NHLS laboratories. The percentage contribution per testing year of the total changed from 28.1% in 2018 to 21.5% in 2021 (Table 1). The number of CrAg tests performed (1.3 million over 4 years) made up between 11.3 and 10.6% of the total CD4 test volumes per annum, with a national average of 10.9%.’

Amended Table 1:

Amended text in data preparation as now analysis was provided by district:

‘The province was populated by the data repository based on the requesting health facility. This information is managed centrally within the NHLS data repository.’

Revised statistical analysis:

‘Statistical analysis

National CD4 and CrAg test volumes were reported per calendar year and month, with the CrAg positivity calculated nationally and per province. Where a province was not captured in the data repository, it was excluded for some analysis. For CrAg- positive samples, the distribution of tests by gender and age categories were analysed. The chi-squared test was used to assess the association between the CrAg result and calendar year, age category and gender, assuming an alpha of 0.05. The monthly test volumes for 2020 and 2021, when lockdown levels were in place, were compared to the corresponding month in 2018 (pre-COVID-19); the percentage change or difference is shown as a line chart, e.g., percentage change between January 2020 and 2018.

The number of SARS CoV-2 cases was reported as a bar chart.[21] This data was used to assign outbreak waves based on the National Institute for Communicable Diseases (NICD) definition (COVID-19 weekly incidence ≥ 30 cases per 100 000 persons) as follows: (i) one: June to August 2020, (ii) two: November 2020 to February 2021, (iii) three: May to September 2021 and (iv) four: December 2021.[22] Data for the months in 2022 during wave four are out of the scope of this study.[23]

For 2020 and 2021 when lockdown measures were introduced, we determined the percentage change in CrAg test volumes by year and month. The highest monthly percentage change in each wave was determined at the national level. We assessed whether the monthly median CD4 (for a count <100 cells/µl) and CrAg positivity differed for two periods defined as pre- (January 2018 to February 2020) and COVID-19 (March 2020 to December 2021). The monthly percentage of specimens with a count <25, 25-50, 51-75 and >75-<100 cells/µl was assessed. Annual CD4 test volumes, irrespective of the count, were assessed to determine the percentage change between 2018 and 2021. This was then compared to the percentage change in reflexed CrAg testing for a CD4 count <100 cells/µl. The contribution of reflexed CrAg to CD4 test was assessed.’

Amended text in data preparation:

‘The data extract was loaded in SAS 9.4 (Cary, NC, USA) for data preparation and analysis. The sample reviewed date was used to extract the year and month for each episode number. The year and month were combined to create a new period variable, e.g., 2018|1 for January 2018. The age-parameter was categorised as: (i) 15-19, (ii) 20-29, (iii) 30-39, (iv) 40-49, (v) 50-59, (vi) 60-69, (vii) ≥70 and (viii) Unknown (where no age was provided). CrAg data is reported only for episodes with a confirmed CD4 <100cells/µl. The CD4 counts <100cells/µl. were further categorised as <25, 25-50, 51-75 and >75-<100 cells/µl. CrAg results were reported as positive or negative. Aggregate CD4 volumes irrespective of count were extracted by calendar year and month.’

Added a new Results heading: ‘Demographics analysis of positive CrAg results:’

Revised Results text:

‘Demographics analysis of positive CrAg results:

Further analysis of CrAg positive data reported no substantial changes in gender distribution per year, with males making up 56% of tests performed (Table 2). An unknown gender contributed <2% of reported results. CrAg positivity for females and males spiked in 2020 at 6.4 and 8.1% respectively (also mimicked in the Unknown gender group). A p-value of ≤0.001 was reported for the chi-squared test for the association of gender with CrAg results (data not shown).

The CrAg positive distribution per age category only showed year-on-year different for those aged 40 to 49 years. CrAg positivity increased from 2018 to 2019, peaking for all age groups in 2020. By 2021, the CrAg positivity for the majority of age groups returned to 2018/19 values except for the 20-29 and 40-49 age groups. A p-value of ≤0.001 was reported for the chi-squared analysis for the association of age category and CrAg results (data not shown).’

Revised table legend:

‘Table 2: Analysis of gender and age category distribution for positive reflexed CrAg test volumes per calendar year, with reference to positivity rates.’

Amended data preparation:

‘Data preparation

Episode data extraction was provided by the NHLS laboratory data repository and included the following variables: (i) episode number, (ii) reviewed date, (iii) age (in years), (iv) gender, (v) province, (vi) CD4 count and (vii) reflexed CrAg result. All rejected and un-reviewed specimens were excluded. The province was populated and managed by the data repository based on the requesting health facility location. The sample reviewed date is electronically generated by the laboratory information system (LIS) when CD4 and CrAg results are verified and released. The age and gender are provided by the health care worker on the laboratory request form and captured on the LIS when specimens are received for testing. The data reported in this paper was not deduplicated due to the absence of a unique patient identifier. Hence, multiple results per patient may be included. An earlier study using de-duplicated patient data reported no significant differences in CrAg positivity, indicating that specimen-level data was sufficient to describe the impact of COVID-19 on episode volumes.[23]

The episode data was uploaded in SAS 9.4 (Cary, NC, USA) for data preparation and analysis. The sample reviewed date was used to extract the year and month for each episode number. The year and month were combined to create a new period variable, e.g., 2018|1 for January 2018. The age parameter was categorised as: (i) 15-19, (ii) 20-29, (iii) 30-39, (iv) 40-49, (v) 50-59, (vi) 60-69, (vii) ≥70 and (viii) Unknown (where no age was provided). CrAg data is reported only for episodes with a confirmed CD4 <100cells/µl. The CD4 counts <100cells/µl. were further categorised as <25, 25-50, 51-75 and >75-<100 cells/µl. CrAg results were reported as positive or negative. The detection rate in the paper is a synonym for CrAg positivity and the terms are used interchangeably. Aggregate CD4 test volumes irrespective of count were extracted by calendar year and month.’

Amended Figure 1 to depict waves.

Amended legend:

‘‘Figure 1: The monthly percentage change in reflexed CrAg test volumes for 2020 and 2021 were compared to 2018 data. The number of SARS CoV-2 cases are reported as bar charts, with waves indicated.’

Amended figure:

’

Amended Results text:

‘Percentage change in CrAg test volumes

The monthly percentage change in total CrAg test volumes relative to the respective month in 2018 ranged from -8.7% (February 2020) to -36.6% (August 2020) (Figure 1). For 2021, the monthly percentage change in CrAg test volumes ranged from -13.3% (December 2021) to -36.1% (August 2021). During the first COVID-19 wave, a monthly percentage change of between -29.1% and -36.6% was reported. For the second wave, a percentage change of between -11.5% and -35.8% was reported. A percentage change of between -26.2% and -36.1% was reported for wave three, with a percentage change of -13.3% for December 2021 (wave four).’

Renamed results headings to:

‘Monthly median CD4 analysis (<100 cells/µl)’

‘Monthly CD4 category analysis (<100 cells/µl)’

Amended provincial analysis as we found a coding error. The number excluded is also now indicated.

Amended Results text:

‘Provincial analysis

The province was not provided for 3 046 samples (0.23%). The comparative percentage change in CrAg test volumes for 2020 versus 2018 at the provincial level ranged from 2.9% (Western Cape) to -33.7% (Free State). Five provinces reported a percentage change in CrAg test volumes of ≤-20% (Table 3). The comparison of 2021 and 2018 CrAg volumes reported a percentage change range of 15.2% (Western Cape) and -35.9% (Gauteng), with five provinces reporting a percentage change of ≤-20%.

In 2018, detection rates ranged from 3.0% (Northern Cape) to 8.2% (KwaZulu-Natal). For 2020, this varied from 3.7% (Northern Cape) to 9.7% (KwaZulu-Natal). Similarly, the detection rate ranged from 3.0% (Northern Cape) to 8.8% (KwaZulu-Natal) for 2021 (Table 3). Between 2018 and 2020, the provincial CrAg detection rate change ranged from 0.2% (Gauteng increased from 5.9% to 6.1%) to 2.4% (Western Cape; increased from 6.3% to 8.7%). Comparing CrAg detection rates between 2018 and 2021, the provincial changes varied from -0.5% (Gauteng) to 1.5% (Western Cape).’

Amended Table 3:

Removed Table 4 – data reported in earlier tables

Amended Table 1 to show the percentage change in Results:

Results text added:

‘The percentage change in CD4 test volumes ranged from -3.3% (2019) to -23.3% (2021) and -4.4% to -27.8% for CrAg.’

Amended Table 1:

Revised limitations section:

‘Limitations

One of the limitations of this study is that only reported specimen-level data for reflexed CrAg testing is reported. By de-duplicating the data, the CrAg positivity would be either slightly higher or lower. However, the former is unlikely to have significantly influenced the outcomes reported here; an analysis of data for KwaZulu-Natal that performed de-duplication noted very minor differences between positivity and prevalence.[23] A further limitation of this study is that there was no linking of the CrAg and COVID-19 results to develop a cohort due to the absence of a unique patient identifier. This cohort analysis would have generated very valuable insights given the differing incubation periods for COVID-19 and cryptococcosis. Another limitation of this study was that no linking of the CrAg and COVID-19 results was undertaken to assess co-infection and the possible impact that COVID-19 infection would have played in the reduction of CD4 count and further immunosuppression that would have led to a higher likelihood of contracting Cryptococcal disease. Data is only reported for reflex CrAg testing, which is in line with local guidelines.[19] The omission of provider-initiated CrAg testing may introduce some bias. However, a local study reported very high coverage of 95% for reflex screening which indicates that any potential bias by omitting provider-initiated data may be very limited.[43] Another limitation was that data was only extracted for testing for those aged 15 years and above. The decision to exclude the younger ages was based on the weekly surveillance data generated by NICD that reported that the cumulative number of COVID-19 cases from 3 March 2020 to 1 January 2022 was 93% for 15 years and older.[44] Additional analysis may be considered to include those aged 10 to 14 years.’

Other minor tracked changes also made.

Updated abstract:

‘Background: Coronavirus disease (COVID-19), caused by the severe acute respiratory syndrome coronavirus 2 (SARS-CoV-2), was first reported in Wuhan, China. Due to the rapid spread globally, it was declared a pandemic in March 2020. Social distancing and lockdown measures were introduced to limit transmission. These strategies could potentially impact the diagnosis and treatment of patients with advanced HIV who are susceptible to secondary infections like cryptococcal disease. In South Africa, reflexed cryptococcal antigenaemia (CrAg) testing and pre-emptive antifungal treatment are recommended preceding antiretroviral therapy initiation for patients with a CD4<100 cells/µl. This study aimed to assess the impact of COVID-19 on CrAg testing in South Africa.

Methods: Specimen-level data was extracted for individuals ≥15 years from the National Health Laboratory Services repository for calendar years 2018 to 2021. Test volumes and CrAg positivity were assessed at national and provincial levels, by age category and gender. The percentage change in annual and monthly CrAg test volumes for 2020 and 2021 (during lockdown levels) are compared to data reported for 2018. The monthly median CD4 and the percentage of samples with a count <25, 25-50, 51-75 and >75-<100 cells/µl were assessed.

Results: Specimen data of 11 944 929 CD4 results included 1 306 456 CrAg tests. Annual CD4 and CrAg test volumes declined by 22.4% and 27.8% for 2020 and 2021 respectively (relative to 2018). There were 23 670 CrAg positive outcomes in 2018 compared to 21 399 (-9.6%) and 17 847 (-24.6%) in 2020 and 2021 respectively. A monthly test volume reduction of up to 36.6%, 35.5%, 36.1% and 13.3% was reported for infection waves one to four. CrAg detection increased from 6.3% in 2018 to 7.5% in 2020. More testing was offered to males (>56%) with a higher detection rate of 8.1% in 2020. Between 81.0% and 81.8% of testing was for patients aged 20 to 49 years. The monthly percentage of specimens <25 cells/µl ranged from 30.2% (June 2019) to 35.3% (August 2020). Overall, the monthly median CD4 ranged from 39 (August 2020) to 45 (March 2019) cells/µl. In 2020, the provincial percentage change in CrAg test volumes ranged from 2.9% to -33.7%.

Conclusion: Our findings confirmed the impact of lockdown measures on both the absolute number of CrAg tests performed and detection (increase in 2020). A smaller impact on the median CD4 was noted. The long-term impact on patient management in immune- compromised individuals needs further investigation.’
