# Supplementary material for: Using laboratory data to assess the impact of coronavirus (COVID-19) on reflex cryptococcal antigenaemia (CrAg) testing in South Africa
Source: PLoS One. 2023 Sep 28;18(9):e0292062. doi: 10.1371/journal.pone.0292062 (PMC10538795; doi:10.1371/journal.pone.0292062)
Supplement: Attachment — Submitted filename: PONE-D-22-31762 Author changes made R3 31 08 2023.docx [file pone.0292062.s004.docx]

**Editorial Changes**

| **Comment** | **Author response** |
| --- | --- |
| 1. Table 1 and 2 headings needs to be shortened | Agreed, Table 1 and 2 heading have been shortened.  Revised text:  ‘Table 1: Annual CD4 and CrAg test volumes, percentage contribution, positivity rate and percentage change (relative to 2018) is reported.’  ‘Table 2: Gender and age category distribution for positive reflexed CrAg samples, with the positivity rates also indicated.’ |
| 2. Provide all IQRs when submitting median values in the manuscript. Please read through the manuscript and correct this e.g. page 9. | Agreed, interquartile ranges have been provided across the manuscript.  Added to abstract:  ‘Overall, the monthly median CD4 ranged from 39 (IQR:15-70)(August 2020) to 45 (IQR:19-72)(March 2019) cells/µl.’  Amended results:  ‘Monthly median CD4 analysis (<100 cells/µl)  Overall analysis of the 4-year test period, reported a median CD4 of 43 (IQR: 18-71), 43 (IQR: 18-71), 43 (IQR: 18-71) and 44 (IQR: 19-72) cells/µl respectively per year (2018 to 2021) (data not shown). Nationally, the monthly median CD4 ranged from 39 (IQR: 15-70)(August 2020) to 45 (IQR: 19-72)(March 2019) cells/µl. For 2018, a monthly median CD4 range of 41 (IQR: 17-69)(December) to 44 (IQR: 19-72)(multiple months) cells/µl was reported (Figure 2). Similarly, the monthly median CD4 ranged from 42 (IQR: 18-71)(January) to 45 (IQR: 19-72)(March) cells/µl for 2019. For 2020 when COVID-19 cases and lockdown measures were introduced, a monthly median CD4 range of 39 (IQR: 15-70) to 45 (IQR: 19-70) was reported. Similarly, for 2021 the monthly median CD4 ranges from 43 (IQR: 19-71) to 45 (IQR: 19-72) cells/µl. When CrAg positivity peaked at 8.0% in April 2020, the median CD4 was 42 cells/µl. The lowest monthly median CD4 reported was 39 cells/µl (IQR: 15-70) in August 2020 with a CrAg positivity of 7.2%. The monthly median CD4 reported a skewness of -1.330031, hence the non-parametric Mann-Whitney test was used (data reported for 48 observations with the exact option used), The results indicated no statistically significant difference between the monthly median CD4 for the two groups (pre-COVID-19 and COVID-19) with a p-value of 0.7431. However, a significant difference was noted for CrAg positivity with a p-value of 0.021.’ |
